# Perineal approach for a gastrointestinal stromal tumor on the anterior wall of the lower rectum

**DOI:** 10.1186/1477-7819-12-62

**Published:** 2014-03-26

**Authors:** Hiroyuki Kinoshita, Yoshifumi Sakata, Yasukazu Umano, Hiromitsu Iwamoto, Kazunari Mori

**Affiliations:** 1 Department of Surgery, Naga Municipal Hospital, 1282, Uchita, Kinokawa, Wakayama 649-6414, Japan

**Keywords:** Gastrointestinal stromal tumor, rectum, perineal approach

## Abstract

**Background:**

Wide margins of resection and regional lymphadenectomy for GIST are not necessary. Several procedures for rectal GIST have been designed according to the location and size of the tumor to preserve the anal function and decrease the morbidity rate.

**Case presentation:**

We report a 61-year-old-man with rectal bleeding. Proctologic examination revealed a small mass of approximately 2 cm in diameter on the anterior wall of the rectum at a distance of 4 cm from the anal verge. Histological examination of the biopsy sample via the rectum led to a diagnosis of GIST due to immunohistochemical positivity for C117 and CD34. Perineal resection was planned because abdominoperineal resection with sacrificing the sphincter function was excessive for this small tumor, and low anterior resection with the double stapling technique was difficult due to the lower position. A hemispheric incision was made from one mid-ischial tuberosity to the other with an apex of approximately 2 cm above the anus. The fascia band and muscles were successively transected in order to expose the anterior wall of the rectum, and excision of the tumor was performed. The postoperative course was uneventful, and the patient remained free from incontinence and recurrence.

**Conclusions:**

This perineal approach for a GIST on the anterior wall of the rectum is one option for preserving the anal function and decreasing the morbidity rate.

## Background

Transanal or transcoccygeal approaches involve common routes for the local excision of rectal tumors. However, these methods are limited to cases of early rectal cancer close to the anal verge without the risk of lymph node metastasis or a non-epithelial tumor at the posterior wall of the rectum. It is difficult to plan the surgical strategy for a gastrointestinal stromal tumor (GIST) at the anterior wall of the lower rectum, as the procedures for the lower rectum are hampered by poor visualization and may cause anal dysfunction or discomfort. Transvaginal excision as described by Hellan is suitable for lesions higher on the anterior wall of the rectum and avoids anal dysfunction [1]. Radical perineal prostatectomy was first described by Young in 1905 [2]. This approach offers a direct route to the apex of the prostate via the external rectal sphincter. We report this method for local excision of a small rectal GIST in a 61-year-old male patient.

## Case presentation

**Figure 1:**
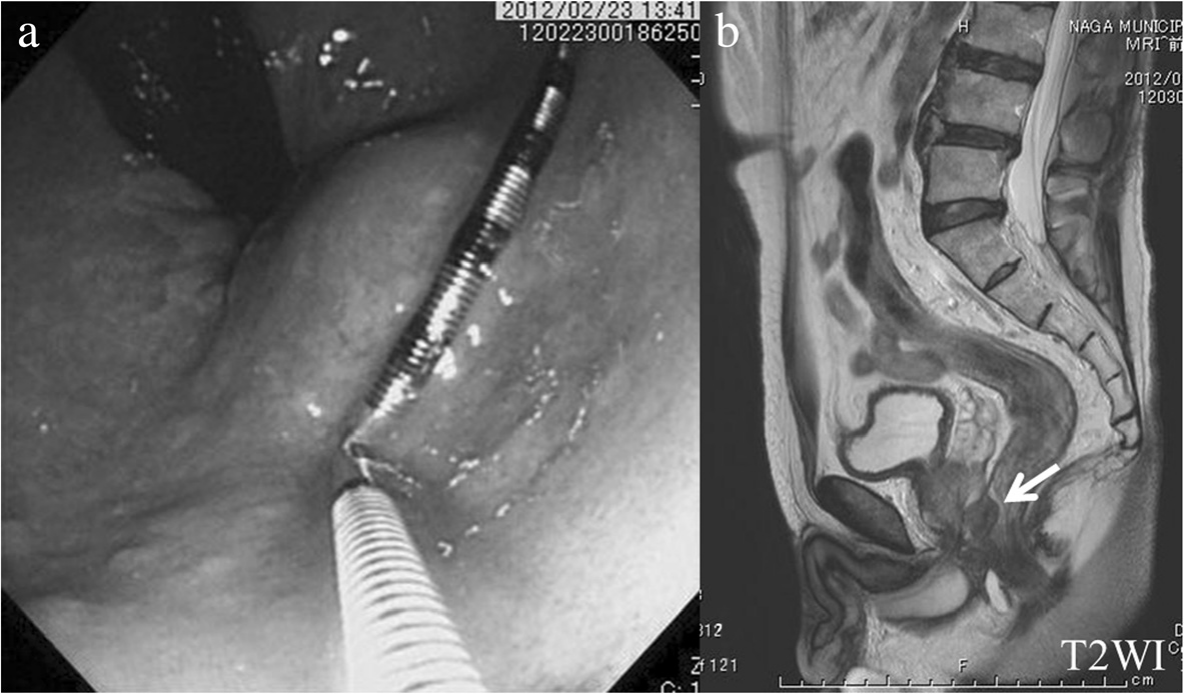
**Colonoscopy and a T2 sagittal magnetic resonance image showed a mass on the anterior wall of the rectum. (a)** Colonoscopy disclosed a bulge which probably originated in the submucosal layer. **(b)** A T2 sagittal magnetic resonance image demonstrated a mass on the anterior wall of the rectum without any invasion to the prostate (arrow).

The patient presented to our hospital with rectal bleeding. He had neither constitutional symptoms nor a history of note. Proctologic examination revealed a small mass of approximately 2 cm in diameter on the anterior wall of the rectum at a distance of 4 cm from the anal verge. The mass was elastic-hard and non-mobile, with a smooth surface. Routine blood tests, serum chemical analysis, and tumor markers showed no abnormalities. Colonoscopy disclosed a bulge which probably originated in the submucosal layer (Figure 1a). A T2 sagittal magnetic resonance image demonstrated a mass on the anterior wall of the rectum without any invasion to the prostate (Figure 1b). Histological examination of the biopsy sample via the rectum led a diagnosis of GIST due to immunohistochemical positivity for C117 and CD34. Perineal resection was planned because abdominoperineal resection with sacrifice of sphincter function was excessive for this small tumor, and low anterior resection with the double-stapling technique was difficult due to the lower position.

**Figure 2:**
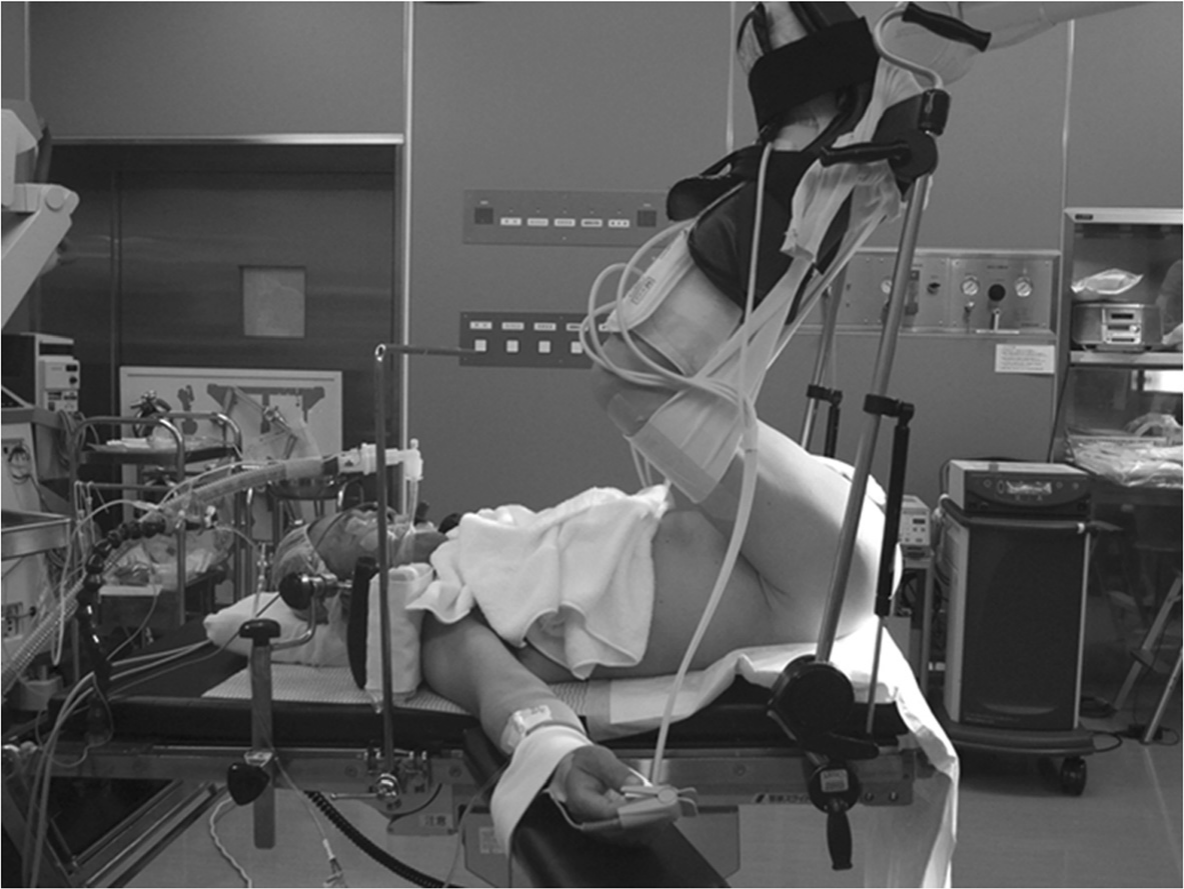
The patient was placed in the exaggerated lithotomy position.

**Figure 3:**
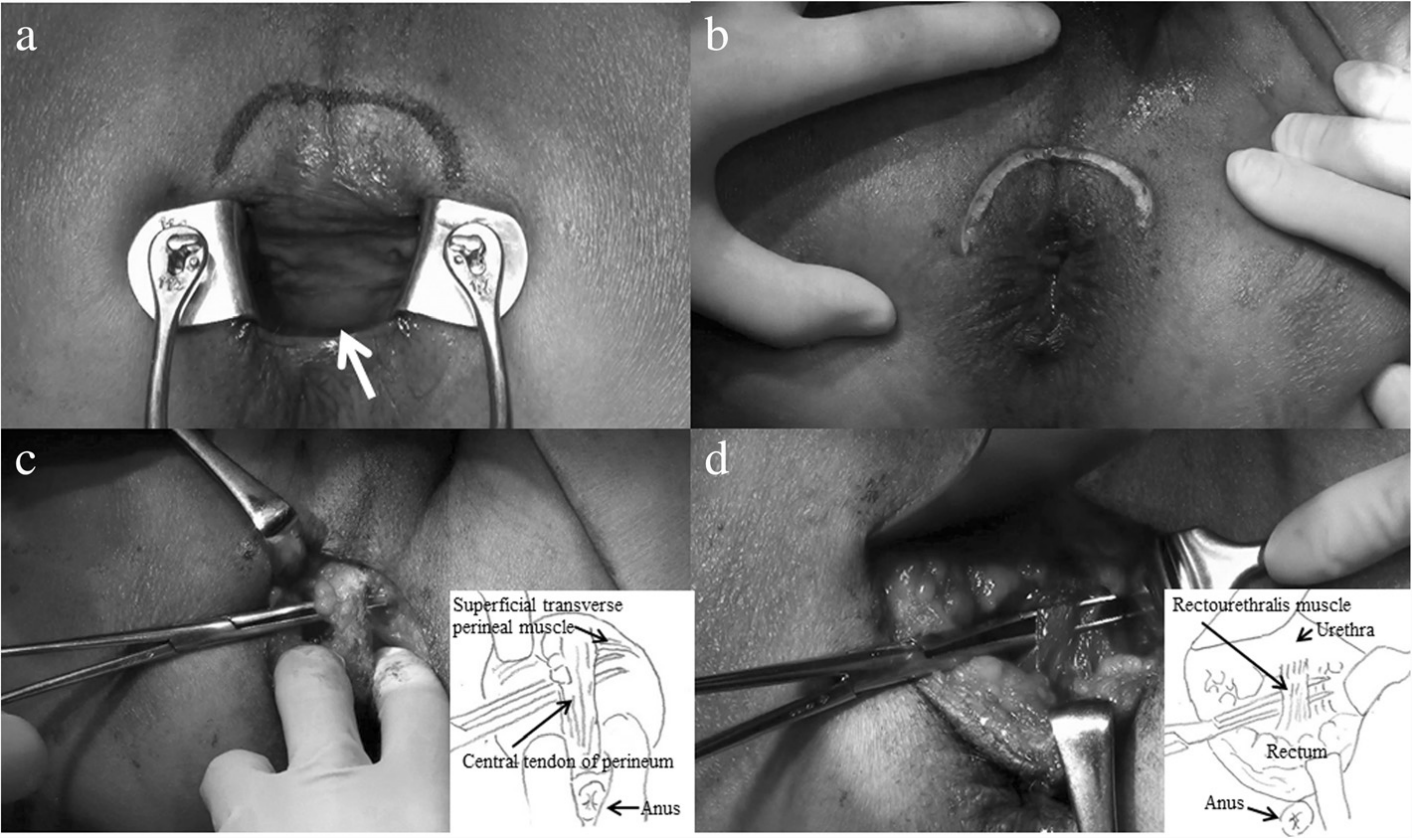
**Operative findings. (a)** The oral edge of the tumor located further from the anal verge than expected with the slack condition of the sphincter (arrow). **(b)** The hemispherical incision was made from one mid-ischial tuberosity to the other with the apex approximately 2 cm above the anus. **(c)** The central tendon was transected after tunneling beneath the superficial transverse perineal muscle. **(d)** The recto-urethral muscle was transected with insertion of an index finger into the rectum to ascertain the tumor.

**Figure 4:**
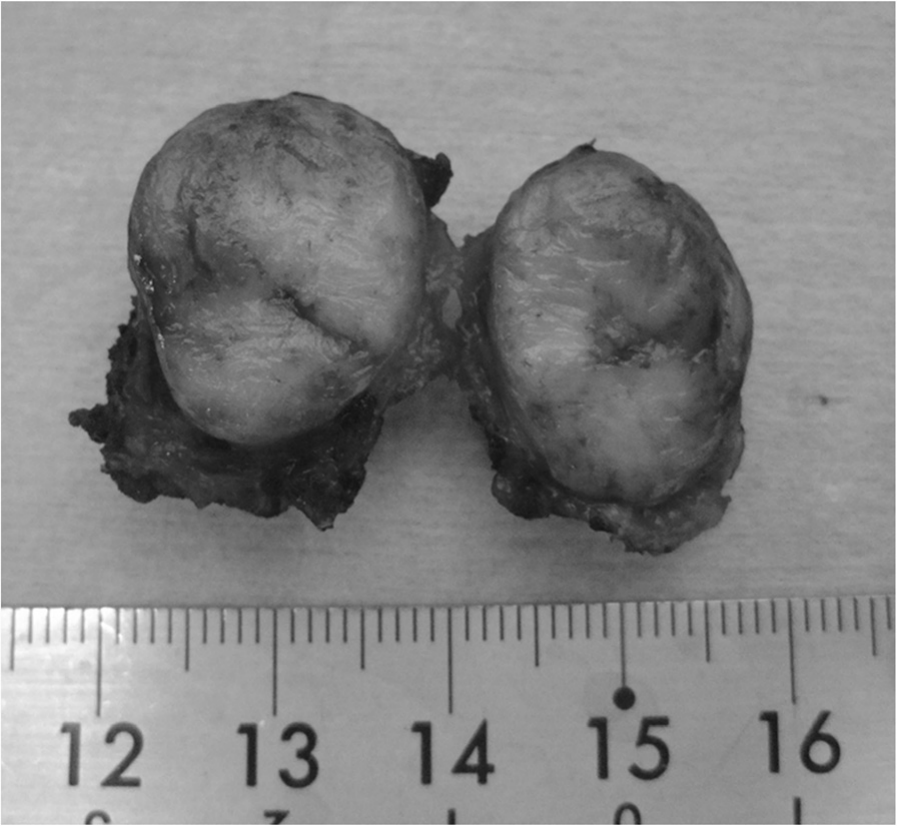
Gross pathological examination of the specimen showed a 2.1 × 2.0 × 1.8 cm fibrous-elastic mass.

Under general anesthesia, the patient was placed in an exaggerated lithotomy position (Figure 2). The oral edge of the tumor was located further from the anal verge than expected with the slack condition of the sphincter (Figure 3a). A hemispheric incision was made from one mid-ischial tuberosity to the other with an apex approximately 2 cm above the anus (Figure 3b). The fascia band was sharply incised and the central tendon of the perineum was transected after tunneling beneath the superficial transverse perineal muscle (Figure 3c). The recto-urethral muscle was confirmed by lifting up the hard catheter passed in the urethra outside the external rectal sphincter in order to prevent urethral injury. Then, this muscle was transected with insertion of an index finger into the rectum to locate the tumor (Figure 3d), and dissection was continued toward the prostate until the oral edge of the tumor was identified. This maneuver exposed the anterior wall of the rectum, and excision of the tumor was performed, using an ultrasonically activated scalpel. The wall of the rectum was closed horizontally with primary suturing. The drain was introduced into an outside layer of the closed rectal wall. A diverting stoma was not performed. Gross pathological examination of the specimen showed a 2.1 × 2.0 × 1.8 cm fibrous-elastic mass (Figure 4). A histopathological report revealed that the tumor consisted of bundle-like proliferations of spindle cells with immunohistochemically positive staining for CD117 and CD34. The mitotic rate was 1 mitotic figure per 50 high-power fields. A diagnosis of GIST, showing ultralow risk behavior, was made.

The postoperative course was uneventful. The patient began a meal on the seventh day. He was discharged on the fourteenth day after the operation. Neither local recurrence nor distant metastasis was noted during follow-up for 18 months.

### Discussion

Surgery has always been the mainstay of GIST treatment, and the prognosis after surgery is influenced by the malignant potential of the tumor and completeness of the primary resection [3,4]. A complete resection with a histologically negative margin is considered a fundamental goal for curative intent. Wide margins and routine lymphadenectomy have not been associated with improved oncologic outcomes [5,6]. However, achieving the complete resection of the GIST located in the lower rectum is difficult because procedures on the pelvic floor are disturbed by poor visualization and the need to preserve sphincter function. Although both the rectum and anus are rare locations, with an incidence of 5% among all GISTs, Miettinen *et al*. reported the treatment of 144 cases of anorectal GIST [7]. In this study the smaller tumors (≤2 cm) were typically treated by enucleation only, excluding one case. Tumors that were >2 cm but ≤5 cm were also usually treated with local excision. Large tumors (>5 cm) were commonly removed by abdominoperineal or anterior resection with impairment of the sphincter function (15 primary cases and 2 cases for the treatment of recurrence).

There are some reports in the literature describing transanal, transcoccygeal, and transvaginal approaches for the local excision of GISTs located in the lower rectum with the aim of decreasing the morbidity rate. Transanal excision is the most minimally invasive; however, there is a limit to the distance from the dentate line. Koscinski *et al*. reported that transanal excision is appropriate for lesions located at an average distance of 3 cm from the dentate line [8]. Furthermore, whether this procedure is possible or not is often dominated by the physique of the patient. Bleday indicated that transcoccygeal excision was especially useful for lesions on the posterior wall of the rectum, and appropriate for lesions located at an average distance of 5 cm from the dentate line [9], but this procedure is associated with a high morbidity rate, such as postoperative fistula occurring in 21% of patients [10]. Transvaginal surgery has a long history. Vorobyov *et al*. demonstrated the excision of a rectal leiomyoma through the vagina [11]. Transanal endoscopic microsurgery (TEM) was first reported by Buess in 1983 [12]. This procedure is primarily used for local excision of selected low, middle, and upper benign rectal tumor via the anus [13]. Although, TEM is a well-established technique, it is unsuitable for lesions close to the anal verge because a set of endoscopic surgical instruments is inserted from the anus.

Radical perineal prostatectomy was first described by Young in 1905 [2]. This procedure was carried out for a patient planned to undergo total prostatectomy without lymphadenectomy [14]. In our case, the tumor was excised with a modification of this method. To our knowledge, only two cases have previously been reported demonstrating this approach for a rectal GIST [15,16]. There are some access routes for local resection of a GIST located in the lower rectum as mentioned above. The indications for these procedures overlap. With regard to our case, transanal or peritoneal resection was planned. The oral edge of the tumor was located further from the anal verge than expected with the slack condition of the sphincter. Then, we finally selected a peritoneal approach. It is thought to be the most important point to maintain the incisional line by palpation of a hard catheter passed into the urethra and locate the tumor with digital examination. This procedure is a safe alternative method for surgeons with abundant experience in abdominoperineal resection for rectal cancer.

## Conclusions

We conclude that this perineal approach for a GIST on the anterior wall of the rectum is one option for preserving the anal function and decreasing the morbidity rate, similar to transanal or transcoccygeal approaches.

## Consent

Written informed consent was obtained from the patient for publication of the report and any accompanying images.

## Abbreviations

GIST: gastrointestinal stromal tumor; TEM: transanal endoscopic microsurgery.

## Competing interests

The authors declare that they have no competing interests.

## Authors’ contributions

All the authors equally participated in preparation of the manuscript. All authors read and approved the final manuscript.
